# Cholic acid as a treatment for cerebrotendinous xanthomatosis: a comprehensive review of safety and efficacy

**DOI:** 10.1186/s13023-025-03889-9

**Published:** 2025-07-29

**Authors:** Gary Pasternack, Jeff Courtney, Gurdyal Kalsi

**Affiliations:** 1 Asklepion Pharmaceuticals, Baltimore, MD USA; 2 Asklepion Pharmaceuticals, LLC, 729 East Pratt Street, Suite 360, Baltimore, MD 21202 USA

**Keywords:** Cerebrotendinous xanthomatosis, Cholic acid, Chenodeoxycholic acid, CYP27A1

## Abstract

Cerebrotendinous xanthomatosis (CTX) is a rare treatable bile acid disorder caused by homozygous or compound heterozygous variants in *CYP27A*, a gene that encodes the mitochondrial enzyme sterol 27-hydroxylase (CYP27A1). CYP27A1 facilitates the production of both cholic acid (CA) and chenodeoxycholic acid (CDCA). Deficiencies in CYP27A1 limit the production of both CA and CDCA, leading to multisystemic cholestanol deposition in membranes, including those of neurons, smooth muscle cells, tendons, and the eye. Because of increased concentrations of cholestanol, a byproduct of cholesterol metabolism, in the brain, cognitive decline develops as a hallmark of CTX. First-line treatment approaches for CTX include off-label prescribed CDCA to reduce serum cholestanol levels. Despite its effectiveness, the success of CDCA administration relies on early diagnosis and low disability scores at the time of initiation. Administration when neurological symptoms arise late in the diagnostic process can lead to worse outcomes, including higher mortality. US Food & Drug Administration-approved CA represents an alternative treatment for CTX. CA reduced cholestanol levels in CSF and blood while also reducing bile acid synthesis and excretion of bile alcohols in the urine. Importantly, outcomes with CA therapy are indistinguishable from those mediated by CDCA therapy and are associated with significantly fewer adverse effects. CA is used as an alternative therapy in patients who cannot tolerate CDCA due to its negative effects. Data from studies on CA strongly support the improvement of liver function, which is likely to be at the crux of secondary pathology, including neurological dysfunction. Because no consensus has been published on the treatment of CTX, Stelten et al (Orphanet J Rare Dis 16:353, 2021) a need exists for a direct comparison of the two approaches.

## Introduction

**Fig. 1:**
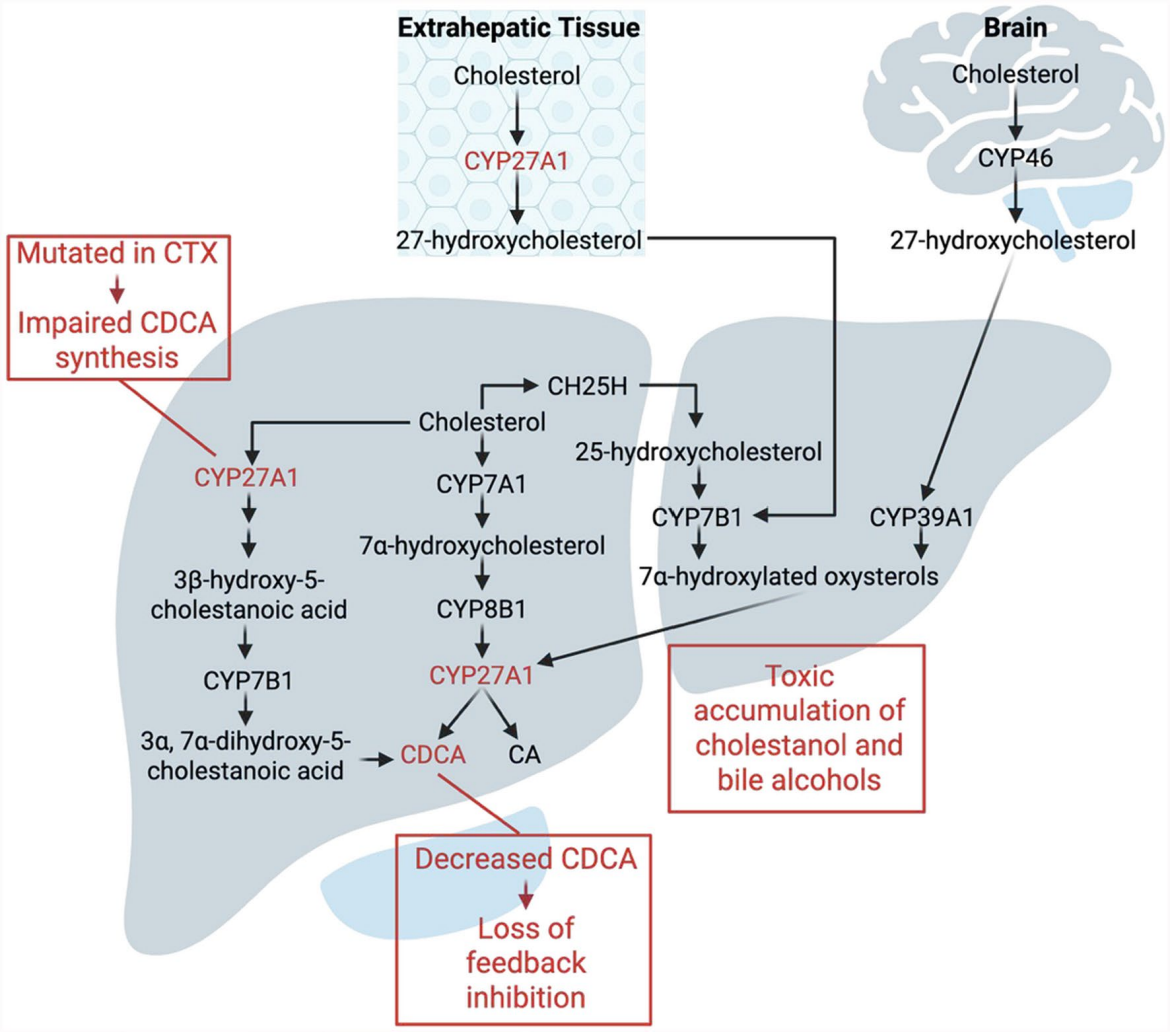
Bile acid synthesis pathways. In the classic pathway, cholesterol is initially converted to 7α-hydroxycholesterol by the rate-limiting enzyme cholesterol 7α-hydroxylase (CYP7A1) in the liver. In the alternative pathway, cholesterol is first converted to oxysterols primarily by sterol 27-hydroxylase (CYP27A1), the first step toward chenodeoxycholic acid (CDCA) synthesis, a process that also occurs in extrahepatic tissues. In the brain, cholesterol is converted to oxysterols by cholesterol 24-hydroxylase (CYP46A1). These oxysterols are then 7α-hydroxylated by oxysterol 7α-hydroxylases CYP7B1 and CYP39A1. While the classic pathway preferentially forms cholic acid (CA), the alternative pathway leads primarily to CDCA formation. In CTX, mutations in CYP27A1 disrupt the acidic bile acid pathway, leading to CDCA deficiency and accumulation of cholestanol and bile alcohols. CYP27A1 sites are highlighted to indicate the enzyme's central role in disease pathogenesis

Cerebrotendinous xanthomatosis (CTX) is a rare treatable bile acid disorder first described in 1937. CTX is caused by homozygous or compound heterozygous variants in *CYP27A*, a gene that is situated on chromosome 2q35 and encodes the mitochondrial enzyme sterol 27-hydroxylase (CYP27A1) [2]. Through the hydroxylation of cholesterol in the alternative bile acid synthesis pathway, CYP27A1 facilitates the production of both cholic acid (CA) and chenodeoxycholic acid (CDCA) (Fig. 1) [3]. Deficiencies in CYP27A1 lead to multisystemic cholestanol deposition in membranes, including those of neurons, smooth muscle cells, tendons, and the eye [4]. Increased concentrations of cholestanol in the brain leads to neuronal cell death, particularly in the cerebellum, and subsequent cognitive decline, a hallmark of CTX. [5]

In addition to progressive neurological dysfunction, CTX is classically distinguished from other lipid storage diseases by adult-onset juvenile cataracts, tendon xanthoma, and pulmonary insufficiency [1,6]. Notably, due to the role of bile acids in digestion, CYP27A1 deficiency is associated with debilitating diarrhea, especially earlier in life, representing the most common catalyst leading to a CTX diagnosis [2]. CTX also produces significant progressive liver disease in children caused by the abnormal bile alcohols produced in lieu of CA. [7–9]

In the healthy system, bile acid production participates in the negative feedback mechanism that regulates bile acid synthesis through repression of the expression of cholesterol 7α-hydroxylase (CYP7A1) and 3-hydroxy-3-methylglutaryl-CoA (HMG-CoA) reductase, and upregulation of the expression of bile salt export pump (BSEP) via farnesoid X receptor (FXR) activation. A disruption in these bile acid synthesis feedback mechanisms accompanies CYP27A1 deficiency. Patients with CTX, therefore, present with elevated serum and urine bile acid alcohols and accumulation of cholestanol caused by compensatory upregulation of CYP7A1, up to 20-fold in some cases [10,11]. Administration of CA and CDCA can restore this negative feedback mechanism to reduce hepatotoxic accumulation of bile alcohols and other symptoms, including, in some cases, neurological dysfunction.

Adapted from Fuchs, et al., 2003. Image prepared by BioRender.

First-line treatment approaches for CTX include off-label prescribed CDCA to improve prognoses by reducing cholestanol levels in serum [12–14]. Apolipoprotein and albumin levels are also reduced in the cerebrospinal fluid (CSF) by CDCA, suggesting a restoration of the integrity of the BBB [15]. The effectiveness of CDCA administration relies on early diagnosis and low disability scores at the time of initiation, an effort hampered by a challenging diagnosis that can be delayed more than 15 years after the onset of symptoms [16,17]. Administration when neurological symptoms arise late in the diagnostic process can lead to worse outcomes, including higher mortality. [16,18,19]

In light of the challenges presented by long-term CDCA therapy, we present support for US Food and Drug Administration (FDA)-approved CA for the treatment of single enzyme deficiencies in bile acid synthesis as an effective alternative bile acid therapy for CTX. In clinical studies, CA reduced cholestanol levels in CSF and blood while also reducing bile acid synthesis and excretion of bile alcohols in the urine. Importantly, outcomes with CA therapy are indistinguishable from those mediated by CDCA therapy [20–22]. and is associated with significantly fewer adverse effects [22]. Indeed, CA is used as an alternative therapy in patients who cannot tolerate CDCA due to its negative effects [22]. Because no consensus has been published on the treatment of CTX [1], a need exists for a direct comparison of the two approaches. Here, we present data that challenge the culture of medicine that does not foster cross-disciplinary consideration of the most efficacious approach to treating a complex, multisystem disease like CTX.

In the sections that follow, we review the major physiological effects of CDCA and CA therapy in individuals with CTX. A significant challenge in drawing conclusions from the findings, especially related to CA administration, is the rare nature of CTX, leading to studies with few patients. Longer-term assessment of bile acid intermediates in the CSF of progressors versus non-progressors is needed as evidence for neurological improvement. However, cholestanol and 7α-hydroxy-4-cholesten-3-one represent important markers for CTX diagnosis and monitoring. Data on CA replacement therapies are, therefore, combined with those from other single enzyme deficiencies to gain interpretative power.

## Urinary excretion of bile alcohols

In addition to elevated plasma and bile cholestanol levels, increased urinary excretion of bile alcohol glucuronides is a biomarker of CTX and other single enzyme deficiencies [12]. In the absence of functioning CYP27A1, for example, intermediates, including 5β-cholestane-3α, 7α-diol and 5β-cholestane-3α, 7α, 12α-triol, fail to be converted to CA and CDCA, respectively. [23–26] Atypical bile acid ratios and accumulation of bile alcohols lead to cytotoxicity and liver fibrosis, further exacerbating hepatic dysfunction [27,28]. Reduced urinary excretion of bile alcohols is a measure of successful treatment of CTX. Additionally, although normalization of elevated cholestanol levels is not always associated with clinical stabilization, follow-up measurements can be useful for dose adjustment of CA.

### Cholic acid

In a Phase 3 open-label, single-arm study of 54 children with single enzyme deficiency (*n* = 5 with CTX; mean age, 3 years old; 18-year follow-up), CA significantly decreased urinary bile alcohols among those in the study (14% post-treatment compared to 72.1% at baseline; *P* < 0.0001), suggesting a reversal of bile alcohol production indicative of abnormal hydroxylation of bile acid intermediates [29]. The continuation study revealed a similar improvement in urinary excretion of bile alcohols over the following 33 months compared to baseline (*P* < 0.05). This finding was echoed by another study that observed a significant decrease in atypical bile acids after 5 years (*n* = 13; *P* < 0.005, compared to baseline) in children with 3β-HSD deficiency [30] and complete disappearance of atypical bile acids after one week of CA treatment [31]. In fact, after changing from ursodeoxycholic or deoxycholic acid to CA due to a lack of efficacy, children with CTX exhibited normal urinary excretion at the last measurement and normalized liver function with results that were clinically indistinguishable from CDCA [32,33]. Notably, a sibling of one of these children who started treatment for CTX with CA remained stable during the 7-year follow-up period [33]. These data corroborated findings from earlier studies that presented children with CTX on CA therapy who exhibited a marked reduction in atypical bile acid production and excretion (statistics not presented) [21,34]. In light of data demonstrating the clinical efficacy of CA that was as good or better than CDCA, CA was recommended for the normalization of bile alcohol excretion. [21]

### Chenodeoxycholic Acid

Atypical bile acid production was reduced in one study of 17 patients with CTX [35]. In a second study of 35 patients with CTX, 29 (82.9%) had urinary excretion of bile alcohols at the initiation of CDCA treatment; all 29 exhibited a significant reduction in bile alcohols (*P* < 0.001; median follow-up, 10.75 years) [13]. These data suggest that CDCA treatment can reverse abnormal hydroxylation of bile acid precursors in the absence of CYP27A1.

### Hepatic effects: liver enzymes, accumulation of toxic metabolites, and hepatotoxicity

Liver function is an important clinical measure of disease progression in CTX. With single enzyme bile acid synthesis deficiencies, atypical bile acids and toxic metabolites (mainly cholestanol) accumulate in the serum [36]. Production of novel bile alcohols also occurs because hydroxylation at C-24 and C-25 proceeds in lieu of C-27 hydroxylation by CYP27A1 [37]. Excessive amounts of gluconated bile alcohols are excreted in the urine and are implicated in intractable diarrhea in patients with CTX [38]. These byproducts of bile acid synthesis deficiencies can lead to cholestasis, cirrhosis, and death if left untreated [39]. Additionally, liver damage secondary to atypical bile acid accumulation leads to increased levels of transaminases in the serum. Alanine transaminase (ALT) and aspartate aminotransferase (AST) generate pyruvic and oxalacetic acids, respectively, in the liver and are elevated in single enzyme deficiencies, including CTX [40]. Therefore, a reduction in these serum transaminases, especially ALT, which is expressed more exclusively in the liver, suggests improvement in liver function.

### Cholic acid

Serum transaminase levels were consistently reduced in studies with CA treatment in children. For example, ALT and AST levels showed significant improvement (*P* < 0.00001, worst compared to best post-treatment values) with CA treatment in a study of 54 children with single enzyme deficiencies, including 3β-hydroxysteroid dehydrogenase (3β-HSD) and CTX [29], AST levels were also significantly improved when the baseline values were compared to the best post-treatment values in a second study that enrolled 41 children (*P* < 0.05, baseline compared to best post-treatment values) [33]. In another study of 13 children with bile acid synthesis defects, CA treatment normalized both ALT and bilirubin levels among patients (*n* = 11 and 12, respectively) with severe cholestasis at baseline (mean follow-up, 6.2 years) with a corresponding reduction in hepatomegaly and steatorrhoea [30,34]. Liver chemistries were also improved in 11/15 (73.3%) of children with 3-β-HSD or 5-β-reductase (statistics not reported) [34]. Further support for the efficacy of CA in treating liver disease associated with CTX comes from the dossier for NDA 205–750, where 3 of 4 patients treated with CA and evaluated for hepatic function were considered responders. Notably, CA is not thought to be cytotoxic in the clinical dosing range due to its hydrophilic face. [41]

Clinical improvement of hepatomegaly, cholestasis, and steatorrhoea was also observed in all 14 children with single enzyme deficiencies on liver biopsy in a prospective observational study at least 5 years after initiation of CA treatment [30]. Cholestasis and liver dysfunction also improved in a second prospective observational study, with 10/15 (66.7%) patients surviving and not requiring liver transplants after 4.5 years. [34] However, there remains a possibility that mild or septal fibrosis may persist after resolution of cholestasis in these patients, which is likely dependent on the amount of time the patient had the condition [31]. Setchell first reported cholestasis as an early manifestation leading to the diagnosis of CTX [9], supporting the relevance of screening for early identification of the disease and increased probability of treatment in children.

### Chenodeoxycholic acid

In a study of 43 patients with CTX, 40 (91%) had normal liver enzymes on treatment. However, 4 patients (9%) had moderate serum liver enzyme elevation that required dose reduction or discontinuation [42]. It should be noted, however, that these data may be complicated by CDCA alone being associated with elevated serum ALT levels. [43]

By virtue of its hydrophobicity, CDCA is cytotoxic [37]. Pediatric patients with CTX (*n* = 1, 0 to < 2 years; *n* = 6, 2 to < 12 years; *n* = 7, 12 to < 18 years) were treated in two non-interventional studies with CDCA. The only infant experienced increased liver function tests that developed within 6 weeks; symptoms normalized after CDCA administration was terminated. However, a caveat to interpretation is the infant had concomitant parechovirus infection and hyperbilirubinemia and received medications with known liver toxicities. [44]

## Neurological effects of bile acids

Bile acids are not synthesized in the central nervous system but exert profound effects on the brain. In a healthy system, bile acids have an established role in neuronal function, including in the modulation of neurotransmission through ionotropic and metabotropic neurotransmitter receptors. The human BASIC channel is more sensitive to CDCA, but not to CA, whereas the voltage-gated large conductance K + (BK) channels are selective for CA rather than CDCA [45,46]. Both CA and CDCA also inhibit N-methyl-D-aspartate (NMDA) and gamma-aminobutyric acid A (GABA_A_) receptor-mediated currents, with CDCA being significantly more potent [47]. The significance of this observation with respect to CTX and other diseases is not presently known.

Conjugated and unconjugated bile acids, including CA and CDCA, cross the blood–brain barrier (BBB) via mechanisms that remain unclear in the healthy system [48]. Conjugated BAs are likely transported through the BBB via organic anion-transporting polypeptides (OATP), apical sodium-dependent bile acid transporter (ASBT), and multidrug resistance protein (MRD) 2 and 4 [49–51]. Similar serum and brain concentrations of unconjugated CA and CDCA suggest these primary bile acids may diffuse freely into the brain. [52,53]

Untreated CTX is associated with seizure and cognitive decline. Cholestane is cleared by the neuron-specific enzyme CYP46A1; however, its expression is low in the cerebellum, a likely cause of cerebellar ataxia in CTX [54]. Although CTX patients characteristically have increased serum cholestanol levels, circulating cholestanol only accounts for a small fraction of the cholestanol in the healthy central nervous system. Instead, 7α-hydroxy-4-cholesten-3-one readily crosses the blood–brain barrier, where it is converted to cholestanol in the brain [55–57]. Increased levels of 7α-hydroxy-4-cholesten-3-one are attributable to a compromised BBB in CTX patients [58]. Administration of exogenous bile acids capable of crossing the blood–brain barrier, including CDCA and CA, represents an effective strategy for preserving neuronal function through restoration of normal bile acid synthetic pathways leading to normalization of intermediate production.

### Cholic acid

The response rate for CA in preventing the progression of neurologic symptoms in CTX cannot be calculated from the available literature, but the effect of CA therapy on cognitive function has been reported. CA therapy was effective in stabilizing the progression of cognitive deterioration in treated patients [20]. The ability of CA to stabilize neurologic function is consistent with the previously described results of Koopman et al*.* [21]*,* and with reduced cholestanol levels in a study of 12 adult patients with CTX [22]. One review article states that only CDCA is capable of stabilizing neurologic disease in CTX. However, the basis of this statement is not clear since the cited references do not provide any data on the results of CA therapy [11]. Additionally, the article fails to acknowledge a recommendation for CA therapy in one of its cited papers. [21]

### Chenodeoxycholic acid

The response rate for CDCA in preventing the progression of neurologic symptoms in CTX varies from approximately 25% to 100% in published studies. The efficacy of CDCA in treating the neurologic manifestations of CTX was first described by Berginer in 1984 [35]. In all 17 patients studied, CDCA appeared to halt disease progression while also improving to varying degrees neurologic function in approximately two-thirds of patients [35]. For example, dementia resolved in 10/13 (76.9%) patients, and pyramidal and cerebellar signs improved or resolved in all 13 patients.

Several other small studies on the effects of CDCA on neurological function cite improvements in ataxia, tremors, and cognitive dysfunction, albeit without specific definitions of how these were measured and few with accompanying statistics [13,42,59]. Improvements in pyramidal and cerebellar signs have also been observed in patients with CTX treated with CDCA (9/15 [60%] and 12/14 [86%], respectively) [35]. Additionally, psychiatric symptoms stabilized, improved, or resolved in between 7.7% and 85.7% in several studies [13,59]. However, the relationship between these findings and mechanisms related to CDCA treatment is unclear.

Cognitive and neurological function improvements have been loosely defined in published studies, and CDCA has produced variable modest results, as expected due to the irreversible nature of neuronal damage observed in CTX. In a study of 28 patients, 20 (71.5%) exhibited cognitive impairment at baseline, and 22 (85%) had cognitive impairment at the last visit (16 were stable, and 6 had worsened) [13]. In the same study, neurological impairment was reported in 20 (77%) at baseline, and 9/20 (45%) exhibited stabilized symptoms and 11/20 (55%) had worsened at the last study visit. Another study reported by the same investigator that enrolled 35 patients showed 1/18 (5.6%) patients with baseline cognitive impairment had improved by the last study visit; the number of patients with neurological impairment had decreased by 3 patients (5%) over the course of the study [13]. Notably, a study of 56 patients with CTX showed early treatment in patients less than 24 years old led to improved, especially among those with good adherences to treatment (*n* = 43). In these patients, neurological symptoms disappeared as measured by the modified Rankin scale and EDSS, whereas among those who initiated CDCA treatment after 24 years old, only 58% and 50% experienced neurological benefits in Rankin and EDSS scores, respectively. [60]

A recent study of 28 patients with CTX used a quantitative approach to measure changes in neurological function (Rankin Scale for Neurological Disability and Expanded Disability Status Scale [EDSS] scoring) [13]. Interestingly, although improvements were noted in some individuals, the mean scores reflected significant worsening from baseline (Rankin and EDSS: *P* = 0.001 and *P* < 0.001, respectively) [13]. Similarly, a study examining 27 CTX patients failed to see objective improvement in CTX patients, although patients apparently improved in self-reported ability to perform activities of daily living [32]. An earlier study of 20 adult patients with CTX reflected the same finding: no objective improvement in cognitive function and pyramidal or cerebellar signs. [32]

*Relationship between neurological effects of CDCA and cholestanol levels.* Cholestanol levels in the serum and CSF has been used, historically, as an indirect measure of neurological damage risk because it accumulates in neurons and may contribute to the brain pathology observed in single enzyme deficiencies [57]. Formation of brain xanthomas due to cholestanol accumulation is thought to cause many of the hallmark neurological symptoms in CTX, including dementia, spinal cord paresis, and cerebellar ataxia. Several studies have reported marked reductions in cholestanol with CDCA treatment. For example, significant reductions were observed in mean cholestanol levels in 14 adult patients (*P* < 0.001) [61] and in a second study of 15 patients with CTX (*P* < 0.005) [59]. However, most studies quantitatively analyze the relationship between serum and CSF cholestanol levels and neurological changes associated with CDCA treatment. [13,15,35,42,59,61–63]

Findings from one of the largest and longest-running studies reported the progression of neurological symptoms in 16 (64%) patients receiving CDCA over the study period. Neurological symptoms were stabilized by CDCA treatment in 6/25 (24%) patients. It is important to note that these investigators found no correlation between cholestanol levels and the progression of neurologic symptomatology, concluding that cholestanol levels were of little predictive value in the context of CDCA treatment [19]. Interestingly, although the relationship between cholestanol remains unclear, an electrophysiology study of one patient with CTX receiving CDCA supported a mechanism involving increased myelination of undamaged axons that could account for improved neurological function [64]. However, additional research is needed to explore this possibility.

## Clinical safety of cholic acid and chenodeoxycholic acid

### Cholic acid

Adverse events (AEs) with CA treatment are rare and generally mild-to-moderate. Notably, most of the clinical studies involved children, whereas the majority of studies with CDCA enrolled adults only. Treatment-emergent AEs (TEAEs) were observed in 2/54 (3.7%) patients in an 18-year phase 3, open-label study and included malaise, jaundice, and skin lesion [29]. In the same study, 4/54 (7.4%) patients discontinued CA treatment; the most common cause of discontinuation was disease progression, and other AEs, which occurred in one patient each, were considered unrelated to treatment [29]. No serious AE (SAEs) related to treatment were observed in this study or in another study that enrolled 15 children with single enzyme deficiencies [30]. A 33-month continuation study showed 8/41 (19.5%) patients discontinuing treatment due to AEs [33]. TEAEs (peripheral neuropathy and nausea) were reported in 2/41 (4.9%) patients but were considered mild and unrelated to CA treatment [33]. A smaller study involving 12 adult patients with CTX showed no adverse events over approximately two years. [22]

### Chenodeoxycholic acid

Conclusions about safety, especially in children, are significantly limited by a lack of systematic presentation of adverse events in publications. In a study of 35 children and adults (42.9% and 57.1%, respectively) with CTX, 74.3% experienced AEs, and 20% had SAEs [13]. In a second study that enrolled 21 patients with CTX, 32.1% experienced SAEs; 16 AEs were also reported in these 9 patients [13]. These limited data suggest markedly lower safety of CDCA compared to CA.

## Discussion

The available data make it difficult to form conclusions about the efficacy of CA relative to CDCA for several reasons. Firstly, CA has been primarily studied in pediatric populations, while CDCA studies have focused mainly on adults. It is clear that among children, CA is safe and well-tolerated; however, conclusions are not possible with CDCA. Caution must, therefore, be used when administering CDCA off-label for CTX, especially in light of possible cytotoxicity that has not been explored in this population. Because early detection of CTX and treatment leads to better outcomes and CDCA administration later in the pathology could be detrimental, bile acid therapy must begin as early as possible. Measurements of cholestasis, as described by Setchell, may create an opportunity for early detection and more successful outcomes in this regard.

The ability of CDCA to arrest or prevent liver disease in CTX patients is difficult to assess due in part to the small number of studies that examined liver function and pathology. Data from studies on CA strongly support the improvement of liver function, which is likely to be at the crux of secondary pathology, including neurological dysfunction. Addressing the cause of CTX and other single enzyme deficiencies at the level of the liver is more likely to produce positive results in terms of preventing the irreversible central nervous system. This is highlighted by studies that measured cholestanol levels and demonstrated a lack of correlation between reduced serum levels and improved cognitive function in patients receiving CDCA [19]. Currently, early diagnosis and treatment initiation in CTX patients is of great importance, as significant reversal of disease progression can be achieved. For this reason, clinical genetic testing is necessary when it comes to patients with an onset of cataracts, chronic diarrhea, and neurological symptoms in early childhood. CA represents an effective alternative with fewer toxicities compared to CDCA.

Importantly, limitations exist in the interpretation of most studies due to the small number and unequal distribution of patients in treatment groups. These factors prevent the ability to control for confounding factors like comorbidities that are common among patients with single enzyme deficiencies [65–67]. The study designs have been largely open-label, single-arm, and non-randomized, further weakening the ability to draw conclusions about treatment effects. For example, open-label studies can also suffer from unintentional patient selection bias. Some studies also have missing data, qualitative assessment of stabilization or lack of disease progression that is not defined in publications, and a lack of statistical analyses [13,68]. Because CTX is rare, clinical trends may represent the best available metric for determining the safest and most efficacious approach to treating the disease (Table 1).

**Table 1:** Summary of clinical studies on cholic acid and chenodeoxycholic acid

Outcome	Dose	Duration of Study	Type of Study	Population	Notable Findings	Reference
*Cholic acid*						
Adverse events	10–15 mg/kg/day	18 years	Phase 3, open-label, single-arm, non-randomized, non-comparative, compassionate treatment	54 children (mean age 3 years at start of treatment) with single enzyme deficiency (*n* = 35, 3-beta-HSD; *n* = 10, 5-beta-reductase; *n* = 5, CTX; *n* = 1, AMACR; *n* = 3, other or unknown; n-31, Zellweger spectrum disorders)	44 AEs in 21/50 (42%) patients, based on analysis of patient records All AEs were mild or moderate	Heubi, et al., 2017 (reporting on CAC-91–10-10) [29]
	500–750 mg/day	798 days average treatment period	NS	12 adult patients (mean age 45.25 years at last visit) with CTX	No AEs observed, including one patient who switched from CDCA to CA due to elevated liver enzymes that normalized while on CA	Mandia, et al., 2019 [22]
	10–15 mg/kg/day	Conducted over 33 months	Phase 3, open-label, single-arm, non-randomized	41 patients (mean age at baseline, 9.0 years; range, 0.1 to 35.6 years) with single enzyme deficiency (*n* = 29) or peroxisomal disorder (*n* = 12), recruited from Study CAC-91–10-10	7 AEs reported	Study CAC-002–001 (unpublished continuation of Heubi, et al., 2017, distinct from Gonzales, et al., 2009)
Adverse events leading to discontinuation	10–15 mg/kg/day	18 years	Phase 3, open-label, single-arm, non-randomized, non-comparative, compassionate treatment	54 children (mean age 3 years at start of treatment) with single enzyme deficiency (*n* = 35, 3-beta-HSD; *n* = 10, 5-beta-reductase; *n* = 5, CTX; *n* = 1, AMACR; *n* = 3, other or unknown; n-31, Zellweger spectrum disorders)	4/54 (7.4%) patients had AEs leading to discontinuation	Heubi, et al., 2017 (reporting on CAC-91–10-10) [29]
	10–15 mg/kg/day	Conducted over 33 months	Phase 3, open-label, single-arm, non-randomized	41 patients (mean age at baseline, 9.0 years; range, 0.1 to 35.6 years) with single enzyme deficiency (*n* = 29) or peroxisomal disorder (*n* = 12), recruited from Study CAC-91–10-10	8/41 (19.5%) discontinued due to AEs unrelated to treatment	Study CAC-002–001 (unpublished continuation of Heubi, et al., 2017, distinct from Gonzales, et al., 2009)
Adverse events related to treatment	10–15 mg/kg/day	18 years	Phase 3, open-label, single-arm, non-randomized, non-comparative, compassionate treatment	54 children (mean age 3 years at start of treatment) with single enzyme deficiency (*n* = 35, 3-beta-HSD; *n* = 10, 5-beta-reductase; *n* = 5, CTX; *n* = 1, AMACR; *n* = 3, other or unknown; n-31, Zellweger spectrum disorders)	3 AEs related to treatment in 2/54 (3.5%) patients TEAEs were malaise, jaundice, and skin lesion	Heubi, et al., 2017 (reporting on CAC-91–10-10) [29]
	10–15 mg/kg/day	Conducted over 33 months	Phase 3, open-label, single-arm, non-randomized	41 patients (mean age at baseline, 9.0 years; range, 0.1 to 35.6 years) with single enzyme deficiency (*n* = 29) or peroxisomal disorder (*n* = 12), recruited from Study CAC-91–10-10	4/41 (9.7%) patients had mild TEAEs	Study CAC-002–001 (unpublished continuation of Heubi, et al., 2017, distinct from Gonzales, et al., 2009)
	10–15 mg/kg/day	18 years	Phase 3, open-label, single-arm, non-randomized, non-comparative, compassionate treatment	54 children (mean age 3 years at start of treatment) with single enzyme deficiency (*n* = 35, 3-beta-HSD; *n* = 10, 5-beta-reductase; *n* = 5, CTX; *n* = 1, AMACR; *n* = 3, other or unknown; n-31, Zellweger spectrum disorders)	3 treatment-related AEs in 2/54 (4%) patients (mean duration of treatment 145 weeks)	Heubi, et al., 2017 (reporting on CAC-91–10-10) [29]
Serious adverse events	10–15 mg/kg/day	18 years	Phase 3, open-label, single-arm, non-randomized, non-comparative, compassionate treatment	54 children (mean age 3 years at start of treatment) with single enzyme deficiency (*n* = 35, 3-beta-HSD; *n* = 10, 5-beta-reductase; *n* = 5, CTX; *n* = 1, AMACR; *n* = 3, other or unknown; n-31, Zellweger spectrum disorders)	28 SAEs were reported No SAEs were related to treatment; disease progression was most commonly noted, followed by diarrhea (3%), dehydration (3%), and urinary tract infection (3%)	Heubi, et al., 2017 (reporting on CAC-91–10-10) [29]
	Mean dose 13 mg/kg at start; mean dose at last dose 6 mg/kg	12.4 years (median follow-up, range 5.6 to 15 years)	Prospective observational	Single enzyme deficiency either 3-beta-HSD or 5-beta-reductase (*n* = 15 children 0.3 to 13.1 years, mediate age at start of therapy 3.9 years)	No SAEs reported CA overdose in 4 patients, but symptoms resolved after dose reduction	Gonzales, et al., 2009 (Trial 2, extension of Trial 1, distinct from CAC-002–001) [30]
Atypical bile acids, bile acid alcohols	10–15 mg/kg/day	10 year follow-up, median follow-up 4.5 years	Prospective observational	15 children with single enzyme deficiency either 3-beta-HSD or 5-beta-reductase	Marked reduction in atypical bile acids (no statistics reported)	Al-Hussaini et al., 2017 [34]
	Mean dose 13 mg/kg at start; mean dose at last dose 6 mg/kg	5 years	Prospective observational	15 children (mean age at initiation of treatment 3.9 years) with 3-beta-HSD (*n* = 13) or with 5-beta-reductase (*n* = 2)	Significant reduction of atypical bile acids at all time points considered in children with 3-beta-HSD (*P* < .005, compared to baseline) Decreased atypical bile acids were reduced in children with 5-beta-reductase (statistics not provided)	Gonzales, et al., 2009 (Trial 2, extension of Trial 1, distinct from CAC-002–001) [30]
	10–15 mg/kg/day	18 years	Phase 3, open-label, single-arm, non-randomized, non-comparative, compassionate treatment	54 children (mean age 3 years at start of treatment) with single enzyme deficiency (*n* = 35, 3-beta-HSD; *n* = 10, 5-beta-reductase; *n* = 5, CTX; *n* = 1, AMACR; *n* = 3, other or unknown; n-31, Zellweger spectrum disorders)	Significant reduction of urinary bile acids (14% compared to 72.1% at baseline with atypical bile acid urinary excretion, *P* < .0001)	Heubi, et al., 2017 (reporting on CAC-91–10-10) [29]
	10–15 mg/kg/day	17 years	Phase 3, open-label, single-arm, non-randomized, non-comparative, compassionate treatment	54 children (mean age 3 years at start of treatment) with single enzyme deficiency (*n* = 35, 3-beta-HSD; *n* = 10, 5-beta-reductase; *n* = 5, CTX; *n* = 1, AMACR; *n* = 3, other or unknown; n-31, Zellweger spectrum disorders)	Significant improvement in urinary bile acid excretion, including in patients with CTX (n = 3) (*P* < .0001)	Heubi, et al., 2017 (reporting on CAC-91–10-10) [29]
	750 mg/day	NS	NS	2 patients on ursodeoxycholic acid; one switched to CA and one to CDCA	Urinary excretion of bile alcohols was suppressed with CA or CDCA (no statistics reported) CA was favored because no adverse effects	Koopman, et al., 1985 [21]
	250 mg TID	44 months	Phase 3, 24-week, randomized, double-blind, placebo-controlled crossover trial	14 adult patients (mean age 39.0 years) with CTX	Bile alcohols (bile 25-tetrol glucuronide) significantly increased (12.5-fold [95% CI: 7.5, 18.9], *P* < 0.0001) following CDCA withdrawal	Kisanuki, et al., 2025 [69]
	10–15 mg/kg/day	Conducted over 33 months	Phase 3, open-label, single-arm, non-randomized	41 patients (mean age at baseline, 9.0 years; range, 0.1 to 35.6 years) with single enzyme deficiency (*n* = 29) or peroxisomal disorder (*n* = 12), recruited from Study CAC-91–10-10	Significant improvement in urinary bile alcohols, baseline compared to best post-baseline analysis (P < .05)	Study CAC-002–001 (unpublished continuation of Heubi, et al., 2017, distinct from Gonzales, et al., 2009)
Cholestanol	500–750 mg/day	798 average treatment period	NS	12 adult patients (mean age 45.25 years at last visit) with CTX	7/7 (100%) treatment-naïve patients had significantly reduced cholestanol with CA treatment (*P* = .028)	Mandia, et al., 2019 [22]
	250 mg TID	44 months	Phase 3, 24-week, randomized, double-blind, placebo-controlled crossover trial	14 adult patients (mean age 39.0 years) with CTX	CDCA withdrawal resulted in 2.8-fold increase 95% CI: 1.5,5.2) in cholestanol	Kisanuki, et al., 2025 [69]
Cholestasis	10–15 mg/kg/day	10-year follow-up, median follow-up 4.5 years	Prospective observational	15 children with single enzyme deficiency either 3-beta-HSD or 5-beta-reductase	Cholestasis was improved (no statistics reported)	Al-Hussaini, et al., 2017 [34]
	Mean dose 13 mg/kg at start; mean dose at last dose 6 mg/kg	21-month study	Non-randomized, open-label, single-arm	21 patients from trial 1 plus 12 new patients (*n* = 33 patients)	No evidence of cholestasis in 4/4 (100%) patients on liver biopsy after CA treatment	Cholbam, US prescribing information, trial 2 (extension of trial 1)
	Mean dose 13 mg/kg at start; mean dose at last dose 6 mg/kg	12.4 years (median follow-up, range 5.6 to 15 years)	Prospective observational	15 children (mean age at initiation of treatment 3.9 years) with 3-beta-HSD (*n* = 13) or with 5-beta-reductase (*n* = 2)	Cholestasis resolved in all 14 patients with liver biopsies at least 5 years after CA treatment	Gonzales, et al., 2009 (Trial 2, extension of Trial 1, distinct from CAC-002–001) [30]
	NS	NS	NS	21 patients with 3-beta-HSD	Cholestasis resolved in some patients, but fibrosis (mild or septal) remained in others	Orphacol EPAR
Liver chemistries	10–15 mg/kg/day	10-year follow-up, median follow-up 4.5 years	Prospective observational	15 children with single enzyme deficiency either 3-beta-HSD or 5-beta-reductase	Normal liver chemistries in 11/15 (73.3%) patients after CA treatment	Al-Hussaini, et al., 2017 [34]
	Mean dose 13 mg/kg at start; mean dose at last dose 6 mg/kg	21-month study	Non-randomized, open-label, single-arm	21 children from trial 1 plus 12 new patients (*n* = 33 total)	Overall response rate of trials 1 and 2 was 44% (2/2 with CTX, 5 other single enzyme deficiencies were also considered) responders (defined as reduced ALT or AST and bilirubin)	Cholbam, US prescribing information, trial 2 (extension of trial 1)
	Mean dose 13 mg/kg at start; mean dose at last dose 6 mg/kg	5 years	NS	15 children (mean age at initiation of treatment 3.9 years) with 3-beta-HSD (*n* = 13) or with 5-beta-reductase (*n* = 2)	Bilirubin and ALT were improved in 12/13 (92.3%) and 11/13 (84.6%) patients, respectively (*P* < .0001)	Gonzales, et al., 2009 (Trial 2, extension of Trial 1, distinct from CAC-002–001) [30]
	10–15 mg/kg/day	18 years	Phase 3, open-label, single-arm, non-randomized, non-comparative, compassionate treatment	54 children (mean age 3 years at start of treatment) with single enzyme deficiency (*n* = 35, 3-beta-HSD; *n* = 10, 5-beta-reductase; *n* = 5, CTX; *n* = 1, AMACR; *n* = 3, other or unknown; n-31, Zellweger spectrum disorders)	ALT and AST levels from baseline were improved relative to baseline (*P* < .0001, worst to best analysis)	Heubi, et al., 2017 (reporting on CAC-91–10-10) [29]
	5–15 mg/kg/day	NS	NS	12 children with single enzyme deficiencies (3-beta-HSD or Δ4-3 oxosteroid 5-reductase)	Liver enzymes normalized within 10 months of initiation of therapy	Potin, et al., 2001 [70]
	10–15 mg/kg/day	33-month study	Phase 3, open-label, single-arm, non-randomized	41 patients (mean age at baseline, 9.0 years; range, 0.1 to 35.6 years) with single enzyme deficiency (*n* = 29) or peroxisomal disorder (*n* = 12), recruited from Study CAC-91–10-10	Statistically improved AST levels when best post-treatment was compared to baseline (*P* < .05) Decreased bilirubin (best post-treatment values compared to baseline, statistics not reported)	Study CAC-002–001 (unpublished continuation of Heubi, et al., 2017, distinct from Gonzales, et al., 2009)
Liver dysfunction	Mean dose 13 mg/kg at start; mean dose at last dose 6 mg/kg	12.4 years (median follow-up, range 5.6 to 15 years)	Prospective observational	15 children (mean age at initiation of treatment 3.9 years) with 3-beta-HSD (*n* = 13) or with 5-beta-reductase (*n* = 2)	In 6/8 (75%) patients who had previously demonstrated a partial response to ursodeoxycholic acid; CA treatment produced resolved jaundice and steatorrhea in all 8 7/8 (87.5%) experienced resolved hepatomegaly Steatorrhea resolved in 9/15 (60%; *P* < .005)	Gonzales, et al., 2009 (Trial 2, extension of Trial 1) [30]
Survival	Mean dose 13 mg/kg at start; mean dose at last dose 6 mg/kg	18-year study (average treatment 310 weeks or 6 years) plus 21-month study	Non-randomized, open-label, single-arm	21 patients from trial 1 plus 12 new patients (*n* = 33 total)	Overall survival for more than 3 years among patients in trials 1 and 2 was 41/62 (67%) 13/41 (32%) survived 10–24 years on treatment	Cholbam, US prescribing information, trial 2 (extension of trial 1)
*Chenodeoxycholic acid*						
Adverse events	750–1000 mg/day (adults); 5–15 mg/kg/day (infants and children)	10.74 years (median duration)	Retrospective cohort	35 patients with CTX; infants/children < 21 years old (*n* = 15) and adults > 21 years old (*n* = 20) (mean age, 36.6 years)	26/35 (74.3%) experienced AEs 3 patients experienced TEAEs (n = 2 constipation, n = 1 toxic hepatitis); none were serious or severe; no recurrence of hepatitis with ongoing treatment at 5 mg/kg/day	Verrips, et al., 2020 (reporting on CDCA-STUK-15–001) [13]
	750 mg/day	5.75 years (median duration)	Retrospective cohort	28 patients with CTX in study CDCA-STUK-15–001 and 25 in CDCA-STRCH-CR-14–001 (mean age 47.4 years)	9/28 (32.1%) experienced AEs; 15 AEs were reported in these 9 patients	Verrips, et al., 2020 (reporting on CDCA-STRCH-CR-14–001) [13]
Serious adverse events	750 mg/day	5.75 years (median duration)	Retrospective cohort	28 patients with CTX in study CDCA-STUK-15–001 and 25 in CDCA-STRCH-CR-14–001 (mean age 47.4 years)	SAEs were reported in 5 patients (17.9%)	Verrips, et al., 2020 (reporting on CDCA-STRCH-CR-14–001) [13]
	750–1000 mg/day (adults); 5–15 mg/kg/day (infants and children)	10.74 years (median duration)	Retrospective cohort	35 patients with CTX; infants/children < 21 years old (*n* = 15) and adults > 21 years old (*n* = 20) (mean age, 36.6 years)	7/35 (20%) patients had SAEs	Verrips, et al., 2020 (reporting on CDCA-STUK-15–001) [13]
Atypical bile acids, bile acid alcohols	750 mg/day	> 1 year of treatment	NS	17 patients with CTX	Abnormal bile acid synthesis was reduced	Berginer, et al., 1984 [35]
	750–1000 mg/day (adults); 5–15 mg/kg/day (infants and children)	10.74 years (median duration)	Retrospective cohort	35 patients with CTX; infants/children < 21 years old (*n* = 15) and adults > 21 years old (*n* = 20) (mean age, 36.6 years)	Significant reduction or normalization in 29/29 (100%) patients with increased urinary excretion of bile alcohols at baseline (*P* < .001)	Verrips, et al., 2020 (reporting on CDCA-STUK-15–001) [13]
Cholestanol or bile acid synthesis intermediates	NS	mean follow-up 5.75 years	Retrospective observational	14 patients with CTX (mean age 29 years)	Statistically significant reduction in serum cholestanol (*P* < .001)	del Mar Amador, et al., 2018 [61]
	750 mg/day	** < cannot access paper > **	** < cannot access paper > **	19 patients with CTX (mean age 32.5 years)	Cholestanol was normalized, but not 7a-hydroxy-cholesten-3-one	Mignarri, et al., 2016 [62] < **cannot access paper** >
	750 mg/day	> 1 year of treatment	NS	17 patients with CTX	Mean plasma cholestanol levels decreased threefold	Berginer, et al., 1984 [35]
	250 mg TID/day (generally)	8 years (mean follow-up)	< 16 investigators collaborated to describe 43 patients >	43 patients with CTX (mean age at diagnosis 33 years)	63% had normal cholestanol levels with CDCA treatment	Duell, et al., 2018 [42]
	125 to 750 mg/day	3-year study	Nationwide Survey	15 patients with CTX (mean age at diagnosis 41 years)	Significantly reduced cholestanol levels (*P* < .0005)	Sekijima, et al., 2018 [59]
	750 mg/day	NS	Prospective observational	3 patients with CTX (mean age 45.7 years)	Cholestanol was decreased	Salen, et al., 1975 [63]
	750 mg/day or 15 mg/kg/day	5.75 (range: 0.0- 25.0 years) years of treatment); 9.0 years (range 0.5–26.3 years) of treatment	Retrospective cohort	28 patients with CTX in study CDCA-STUK-15–001 and 25 in CDCA-STRCH-CR-14–001 (mean age 47.4 years)	Cholestanol and 7a-hydroxy-cholesten-3-one were improved (*P* < .001) Significance was consistent between patients < 21 or 21 and over years when treatment was initiated	Verrips, et al., 2020 (reporting on CDCA-STRCH-CR-14–001) [13]
	750–1000 mg/day (adults); 5–15 mg/kg/day (infants and children)	10.74 years (median duration)	Retrospective cohort	35 patients with CTX; infants/children < 21 years old (*n* = 15) and adults > 21 years old (*n* = 20) (mean age, 36.6 years)	Significant reduction of mean serum cholestanol levels compared to baseline in both patient groups (*P* < .001) at all visits The timing of these visits after the first treatment is unclear and data were missing from 25% of patients (adult and infant/children < 21 years old)	Verrips, et al., 2020 (reporting on CDCA-STUK-15–001) [13]
	750 mg/day	5.75 years (median duration)	Retrospective cohort	28 patients with CTX in study CDCA-STUK-15–001 and 25 in CDCA-STRCH-CR-14–001 (mean age 47.4 years)	Diarrhea improved or disappeared in 64.3% of patients	Verrips, et al., 2020 (reporting on CDCA-STRCH-CR-14–001) [13]
Diarrhea	750 mg/day	5.75 years (median duration)	Retrospective cohort	28 patients with CTX in study CDCA-STUK-15–001 and 25 in CDCA-STRCH-CR-14–001 (mean age 47.4 years)	Diarrhea improved or disappeared 64.3%; or 14/26 or 54% had diarrhea at baseline and 42% or 11/26 still had diarrhea at the most recent clinical visit	Verrips, et al., 2020 (reporting on CDCA-STRCH-CR-14–001) [13]
	750–1000 mg/day (adults); 5–15 mg/kg/day (infants and children)	10.74 years (median duration)	Retrospective cohort	35 patients with CTX; infants/children < 21 years old (*n* = 15) and adults > 21 years old (*n* = 20) (mean age, 36.6 years)	Diarrhea resolved in 100% of patients who had diarrhea (23/31; 74%)	Verrips, et al., 2020 (reporting on CDCA-STUK-15–001) [13]
	250 mg TID/day (generally)	8 years (mean follow-up)	< 16 investigators collaborated to describe 43 patients >	43 patients with CTX (mean age at diagnosis 33 years)	91% had normal liver enzymes on treatments 9% had moderate liver enzyme elevation that required dose decrease or discontinuation	Duell, et al., 2018 [42]
Liver chemistries	125 to 750 mg/day	3-year study	Nationwide Survey	15 patients with CTX (mean age at diagnosis 41 years)	Liver injury in 3 patients	Sekijima, et al., 2018 [59]
Liver dysfunction	750 mg/day	> 1 year of treatment	NS	17 patients with CTX	Dementia resolved in 10/13 (76.9%) patients Pyramidal signs resolved in 5/5 (100%) Cerebellar signs resolved or improved in 5/13 (38.5%) and 8/13 (61.5%), respectively Peripheral neuropathy resolved in 6/7 (85.7%)	Berginer, et al., 1984 [35]
Neurological function	NS	mean follow-up 5 years	Retrospective observational study	14 patients with CTX (mean age 29 years)	Mean EDSS scores improved in 4 (28.6%) stable in 5 (35.7%,) and worse in 5 (35.7%)—the ones who had worsened by at least 1 point (n = 3) had the longest interval between symptoms and treatment, cognitive impairment similar to pivotal study (not defined) was not reported, psychiatric impairment was not reported	del Mar Amador, et al., 2018 [61]
	250 mg TID/day (generally)	8 years (mean follow-up)	NS	43 patients with CTX (mean age at diagnosis 33 years)	57% had improved and stabilized disease (no definitions provided) 7 (20%) had disease progression (all > 25 years old with significant neurological dysfunction)	Duell, et al., 2018 [42]
	NS	NS	Retrospective case series, nationwide (Spain)	25 patients with CTX	6/25 (24%) received CDCA; 18 (72%) received CDCA plus a statin 25/25 (100%) had decreased or normalized serum cholestanol 28% remained stable (1 CDCA, 6 CDCA plus statin) 14/25 (5 with CDCA, 9 with CDCA plus statin) declined 5/25 (20%) died during the follow-up period, albeit were significantly older than those who survived (*P* < .001) No clinical improvement was observed despite normalized cholestanol	Pilo-de-la-Fuente, et al., 2011 [19]
	750–1000 mg/day (adults); 5–15 mg/kg/day (infants and children)	10.74 years (median duration)	Retrospective cohort	35 patients with CTX; infants/children < 21 years old (n = 15) and adults > 21 years old (n = 20) (mean age, 36.6 years)	Pyramidal dysfunction stabilized or improved in 9/15 (60%) patients Cerebellar dysfunction stabilized or improved in 12/14 (86%) patients Parkinsonian symptoms did not change in 2/2 (100%) patients	Pivotal study CDCA-STUK-15–001
	125 to 750 mg/day	3-year study	Nationwide Survey	15 patients with CTX (mean age at diagnosis 41 years)	Improved tremor in 2/15 (13.3%) Reduced ataxia in 1/15 (7.7%), Improved cognitive dysfunction in 1/15 (7.7%) Reduced psychiatric symptoms in 1/15 (7.7%)	Sekijima, et al., 2018 [59]
	750–1000 mg/day (adults); 5–15 mg/kg/day (infants and children)	10.74 years (median duration)	Retrospective cohort	28 patients with CTX in study CDCA-STUK-15–001 and 25 in CDCA-STRCH-CR-14–001 (mean age 47.4 years)	Psychiatric impairment resolved, improved or stabilized in 12/13 (92.3%) patients	Verrips, et al., 2020 (reporting on CDCA-STRCH-CR-14–001) [13]
	750 mg/day	5.75 years (median duration)	Retrospective cohort	28 patients with CTX in study CDCA-STUK-15–001 and 25 in CDCA-STRCH-CR-14–001 (mean age 47.4 years)	Stable Rankin and EDSS scores were stable in 61.5% and 50%, respectively, but no definition of stable 80% of patients were in these categories Statistically significant worsening of mean scores (Rankin and EDSS) from baseline was observed (*P* = .001 and *P* < .001, respectively)—people in this study were on average older and had higher disability scores at baseline	Verrips, et al., 2020 (reporting on CDCA-STRCH-CR-14–001) [13]
	750 mg/day	5.75 years (median duration)	Retrospective cohort	28 patients with CTX in study CDCA-STUK-15–001 and 25 in CDCA-STRCH-CR-14–001 (mean age 47.4 years)	20/28 (71.5%) patients had cognitive impairment at baseline 22/28 (85%) patients had cognitive impairment at last visit (16 or 73% were stable, 6 or 27% had worsened) Psychiatric impairment was observed in 13/26 (50%) of patients at baseline At the most recent study visit, 1 (7%) had stabilized and 12 (92%) had worsened 20 (77%) had neurological impairment at baseline 9/20 (45%) patients stabilized at last visit 11/20 (55%) patients worsened at last study visit	Verrips, et al., 2020 (reporting on CDCA-STRCH-CR-14–001) [13]
	750–1000 mg/day (adults); 5–15 mg/kg/day (infants and children)	10.74 years (median duration)	Retrospective cohort	35 patients with CTX; infants/children < 21 years old (n = 15) and adults > 21 years old (n = 20) (mean age, 36.6 years)	18/31 (58%) had cognitive impairment at baseline 16/31 (51.6%) had cognitive impairment at last study visit 1/18 (5.6%) patient had improved 15/18 (94%) were stable 20/31 (65%) had neurological impairment at baseline 17/31 (54.8%) at most recent study visit No definitions provided, cognitive impairment was not defined	Verrips, et al., 2020 (reporting on CDCA-STUK-15–001) [13]
	750–1000 mg/day (adults); 5–15 mg/kg/day (infants and children)	10.74 years (median duration)	Retrospective cohort	35 patients with CTX; infants/children < 21 years old (n = 15) and adults > 21 years old (n = 20) (mean age, 36.6 years)	Polyneuropathy stabilized or improved in 11/11 (100%) patients Epilepsy resolved in 3/3 (100%) with seizures	Verrips, et al., 2020 (reporting on CDCA-STUK-15–001) [13]
	750–1000 mg/day (adults); 5–15 mg/kg/day (infants and children)	10.74 years (median duration)	Retrospective cohort	35 patients with CTX; infants/children < 21 years old (n = 15) and adults > 21 years old (n = 20) (mean age, 36.6 years)	Psychiatric impairment resolved, improved, or stabilized 5/6 (85.7%)	Verrips, et al., 2020 (reporting on CDCA-STUK-15–001) [13]
	750–1000 mg/day (adults); 5–15 mg/kg/day (infants and children)	10.74 years (median duration)	Retrospective cohort	35 patients with CTX; infants/children < 21 years old (n = 15) and adults > 21 years old (n = 20) (mean age, 36.6 years)	Rankin Score improved in 4/26 (15%) and stabilized in 18/26 (69%), and deteriorated in 4/26 (15%) EDSS improved in 6/26 (23%), stabilized in 14/26 (54%), and worsened in 6/26 (23%) Mean Rankin and EDSS decreased from baseline to the most recent study visit, but not statistically significant	Verrips, et al., 2020 (reporting on CDCA-STUK-15–001) [13]
	750 mg/day	NS	NS	20 patients with CTX (range, 26–71 years old)	No objective improvement in cognitive function and cerebellar or pyramidal signs Self-reported improvement in activities of daily living in 10/20 (50%) patients	Waterreus, et al. 1987^32^
	750–1000 mg/day (adults); 5–15 mg/kg/day (infants and children)	10.74 years (median duration)	Retrospective cohort	35 patients with CTX; infants/children < 21 years old (n = 15) and adults > 21 years old (n = 20) (mean age, 36.6 years)	8/31 (26%) had xanthomas at baseline 10/31 (32%) had xanthomas at the most recent clinical visit	Pivotal study CDCA-STUK-15–001
Xanthoma	750 mg/day	5.75 years (median duration)	Retrospective cohort	28 patients with CTX in study CDCA-STUK-15–001 and 25 in CDCA-STRCH-CR-14–001 (mean age 47.4 years)	21/26 (81%) had xanthomas at baseline 15/21 (71%) were stable 6/21 (29%) had worsened at the most recent clinical visit	Supportive study CDCA-STRCH-CR-14–001

3-beta-HSD, 3-beta-hydroxysteroid dehydrogenase deficiency; AE, adverse event; ALT, alanine transaminase; AMACR, alpha-methylacyl-CoA racemase; AST, aspartate aminotransferase; CA, cholic acid; CDCA, chenodeoxycholic acid; CSF, cerebrospinal fluid; CTX, cerebrotendinous xanthomatosis; EDSS, expanded disability status score; NS, not specified; SAE, serious adverse event; TEAE, treatment emergent adverse event

## Data Availability

N/A.
